# Analysis of mortality of users treated at a Mobile Emergency Care Service: an observational study, Paraná, 2019-2020

**DOI:** 10.1590/S2237-96222025v34e20240092.en

**Published:** 2025-09-15

**Authors:** Erika Fernanda dos Santos Bezerra Ludwig, Maria do Carmo Fernandez Lourenço Haddad, Mariana Ângela Rossaneis Moreira, Aroldo Gavioli, Lilia de Souza Nogueira, Eleine Aparecida Penha Martins

**Affiliations:** 1 Universidade Estadual de Londrina, Programa de Pós-Graduação em Enfermagem, Londrina, PR, Brazil; 2 Hospital Universitário Regional de Maringá, Enfermagem, Maringá, PR, Brazil; 3 Escola de Enfermagem da Universidade de São Paulo, Programa de Pós-Graduação em Enfermagem, São Paulo, SP, Brazil

**Keywords:** Mortality, Emergencies, Emergency Medical Services, Ambulances, Survival Analysis, Mortalidad, Emergencias, Servicios Médicos de Emergencia, Ambulancias, Análisis de supervivencia

## Abstract

**Objective:**

To analyze the mortality of users attended by a Mobile Emergency Care Service located in Paraná.

**Methods:**

This is a cross-sectional analytical study based on service reports from 2019 and 2020. The Mobile Emergency Care Service covered 21 municipalities, divided into hubs A and B. The dependent variables defined were death and time of care. Survival functions were calculated using the Kaplan-Meier estimator and the log-rank test; hazard ratio (HR) for death, by Cox regression with 95% confidence interval (95%CI).

**Results:**

The study evaluated 13,326 instances of care provided. Of these, 246 resulted in death. The risk of death was higher in time-sensitive calls (HR 0.17; 95%CI 0.008; 0.37), in 2020 (HR 2.09; 95%CI 1.39; 3.16), attended by advanced support (HR 21.51; 95%CI 12.61; 36.70) and in Hub B (HR 4.26; 95%CI 2.53; 7.17).

**Conclusion:**

Mortality was higher in cases that had a long wait for time-sensitive calls, occurred in less populated regions, and were dealt with by advanced support in 2020.

## Resumen

**Objetivo:**

Analizar la mortalidad de usuarios atendidos por un Servicio de Atención Móvil de Urgencias ubicado en Paraná.

**Métodos:**

Se trata de un estudio analítico de corte transversal desarrollado con base en reportes de servicios de los años 2019 y 2020. El Servicio de Atención Móvil de Urgencias abarcó 21 municipios, regionalizados en los polos A y B. Las variables dependientes definidas fueron fallecimiento y tiempo de atención. Las funciones de supervivencia se calcularon utilizando el estimador de Kaplan-Meier y la prueba de log-rank; hazard ratio (HR) de muerte, mediante regresión de Cox con intervalo de confianza del 95% (IC95%).

**Resultados:**

Se analizaron 13.326 consultas. De estos, 246 resultaron en muerte. El riesgo de muerte fue mayor en las solicitudes urgentes (HR 0,17; IC95% 0,008; 0,37), en 2020 (HR 2,09; IC95% 1,39; 3,16), atendidas con apoyo avanzado (HR 21,51; IC95% 12,61; 36,70) y en el polo B (HR 4,26; IC95% 2,53; 7,17).

**Conclusión:**

La mortalidad fue mayor en las atenciones que tuvieron un intervalo de tiempo prolongado por razones sensibles al tiempo, ocurridas en regiones menos pobladas y atendidas con apoyo avanzado en 2020.

**Ethical aspects**

This research respected ethical principles, having obtained the following approval data:

Research Ethics Committee: Universidade Estadual de Londrina

Opinion number: 4.350.880

Approval date: 20/10/2020

Certificate of Submission for Ethical Appraisal: 39011920.1.0000.5231

Informed Consent Form: Exempted.

## Introduction

On the global stage, the organization of emergency care has been one of the challenges faced by health managers (1). In 2019, the The World Health Assembly highlighted the importance of emergency care systems as an essential component of health coverage. Member countries were encouraged to assess their policies and needs in this area of action, given its decisive influence on the results of the entire health system (2,3).

Countries that have organized their emergency care systems have had positive impacts in terms of reducing morbidity and mortality, time, and cost of care, especially in situations of trauma and chronic degenerative diseases (4,5). These conditions are part of the priority care lines of the Emergency Care Network of the Unified Health System and are time-sensitive, being influenced by the quality of pre-hospital care, that is, by the Mobile Emergency Care Service (Serviço de Atendimento Móvel de Urgência, SAMU) (6).

Implemented in 2003 in Brazil, SAMU aims to reduce mortality, the length of hospital stay and sequelae and other adverse situations resulting from late interventions (7). The agility in the response between the request for help and the care for clinical and traumatic injuries is considered important in ensuring the survival of the population served (8).

Despite the advances made by SAMU, which covered 85.0% of the Brazilian population in 2022, there are still several factors that interfere with the time for care and, consequently, the survival of people served by the service. Among these factors, regional disparities stand out, influenced by the organization of the health network and local resources (9).

The number of services provided by SAMU in Brazil had a sustained and significant increase between 2015 and 2019, surpassing the growth rates of the covered population and distributed resources. This fact, also observed in other countries, is related to longer response times and delays in serving users with serious health issues, negatively impacting the survival of the population served and compromising the indicators of the health system as a whole (10).

Given the need to promote equity of access and quality of care offered by pre-hospital care, this study aims to analyze the mortality of users attended by a SAMU located in the interior of Paraná. It is assumed that the process of regionalization and organization of the pre-hospital network may affect the response time and impact the survival of the population served.

## Methods

### Study design

This is a cross-sectional, analytical, and documentary study, conducted between January 1, 2019, and December 31, 2020, based on reports of care provided by first responders who provided care at a SAMU in the interior of Paraná.

### Context

The SAMU Emergency Regulation Center was implemented in 2004 and regionalized in 2012. The center covered 970 thousand inhabitants distributed across 21 municipalities, organized into two service hubs, called A and B. 

**Figure 1:**
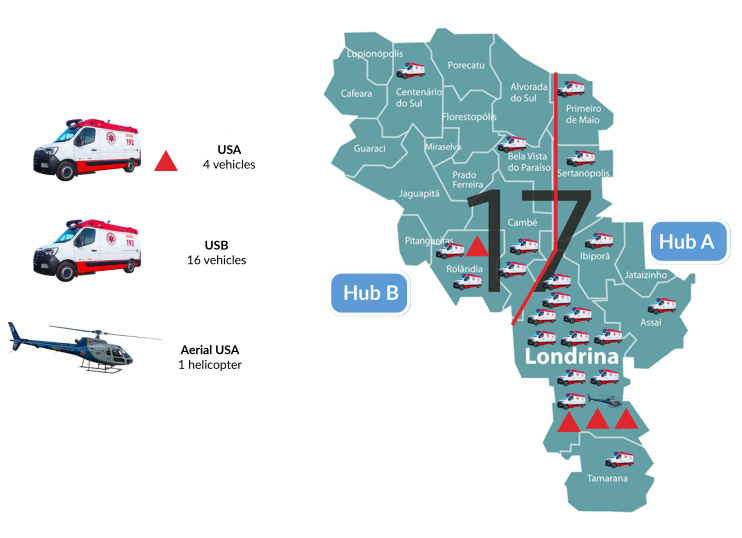
Distribution of mobile resources, advanced support unit (ASU) and basic support unit (BSU) of the Mobile Emergency Care Service under study, according to regionalization in service centers. Paraná, 2024

Hub A comprised seven municipalities, with 696,030 people, with three advanced support units, one rotary wing air medical unit and six basic support units. Hub Bcomprised 14 municipalities, with 273,970 people, and had an advanced support unit and five basic support units, distributed across four decentralized bases (11) (Figure 1).

The distribution of vehicles across the entire area of the 21 municipalities, the regionalization into Hubs A and B and the location of decentralized bases with the available mobile resource were observed (Figure 1). Advanced support services at Hub A could come from seven municipalities, which were described below, along with the approximate distance from the service base, broken down in kilometers (km): Londrina (municipality with advanced support unit base), Ibiporã (21.00 km), Jataizinho (26.70 km), Sertanópolis (45.30 km), Assaí (47.40 km), Tamarana (54.00 km) and Primeiro de Maio (69.00 km). In this Hub, except for Jataizinho, served by the Ibiporã basic support unit, all the others had a basic support base (11).

In Hub B, emergency calls could originate from 14 municipalities, including: Rolândia (municipality with advanced support unit base), Cambé (10.80 km), Jaguapitã (34.80 km), Prado Ferreira (37.00 km), Pitangueiras (42.80 km), Miraselva (48.70 km), Bela Vista do Paraíso (51.80 km), Guaraci (56.00 km), Florestópolis (58.60 km), Centenario do Sul (69.00 km), Porecatu (62.00 km), Alvorada do Sul (78.90 km), Lupionópolis (81.00 km), and Cafeara (87.50 km). In this Hub, four of the 14 municipalities had a base with a basic support unit, with Rolândia being responsible for serving Jaguapitã and Pitangueiras for services with basic resources. Centenário do Sul provided assistance to other municipalities without their own base (11).

This service operates continuously, 24 hours a day. To ensure the response time, understood as the interval between the request to telephone number 192 and the arrival of the team on site to provide assistance, the coordination and agility of several professionals were essential. The medical regulation assistant telephone operator received requests via telephone number 192, noted down the victim’s personal data and location, and transferred the call to the regulating doctor. This would direct questions to the applicant, making it possible to estimate the level of priority (emergency, urgent, not urgent) of the service, defining the mobile resource to be dispatched to the location of the incident. Subsequently, the radio operator conducted the dispatch by contacting the mobile resource team to begin its trip. The driver’s actions were equally crucial, as he had to identify the quickest and safest route to ensure the team’s arrival at the service location. 

All these steps were exposed to possible failures that could compromise response time, such as incorrect address, inadequate choice of mobile resource according to the victim’s needs, insufficient number of vehicles to meet the demand for requests or improper definition of the route to the location of the incident (3).

### Participants

Only those services that generated medical regulation and dispatch of teams for assistance were considered, since the report was only completed in cases where the mobile resource was moved. Reports with incomplete data and calls generated solely to confirm death were excluded, since the objective of this study was to analyze mortality considering the actions of SAMU pre-hospital care.

### Variables

Data were collected between January and December 2021, in accordance with the established inclusion criteria. An instrument developed by the researcher using Microsoft Excel was used. Data could be accessed by the researcher and two additional people, participants in the collection and duly trained, to guarantee double verification of the collected data. 

The dependent variables death and response time were collected. The first variable was considered during pre-hospital care and categorized into two classes: death and non-death. The response time was determined from the request made on telephone number 192 until arrival at the location for assistance, measured in minutes.

The independent variables analyzed were sex, age categorized in years (0-11, 12-19, 20-59 and ≥60 years), as recommended by the World Health Organization, municipality of origin of the service, nature of the service (assistance, transfer), reason for the request (external causes, cardiac, neurological, respiratory, psychiatric or others), day of the week and shift of service (morning, afternoon, evening, dawn), type of team dispatched (basic support unit, advanced support unit) and year in which the service was provided (2019, 2020).

### Data sources and measurement

The service reports from the two municipalities that had advanced support units were selected – this is a more specialized resource aimed at users in critical condition, that is, those who needed agility upon arrival at the location. This selection priviledged the coverage area of hubs A and B, which guaranteed the analysis of the 21 municipalities served by SAMU. 

### Study size and bias control

The sample size considered the total number of services provided by SAMU in the period studied, with 230,765 calls requiring dispatch of resources to the requested location. To achieve numerical representativeness that would allow generalization, the sample was calculated using the PEPI software (Programs for Epidemiologists, version 4.0), in addition to a formula for estimating proportions, with an acceptable margin of error of 1% and a confidence level of 95%, which established a minimum sample of 9,221 reports of emergency care.

Data were collected until the minimum sample was reached. Many reports presented partial incompleteness of data. 13,326 reports were analyzed, of which 9,876 were filled out, representing 5,199 (52.64%) services from Hub A and 4,677 (47.35%) from Hub B.

### Statistical methods

For data analysis, a significance level of 5% was considered (p-value>0.05). Survival functions were calculated using the Kaplan-Meier estimator, with and without stratification, using the log-rank test and the Breslow global comparison (generalized Wilcoxon). For the analysis of the risk of the hazard ratio (HR) of death, depending on the time, the univariate regression model (Cox regression) was used. Pearson’s chi-square test was used to calculate the death rate (per 1,000 visits) in association with time (considered from the request made on telephone number 192 until arrival at the location for care) and the care center.

## Results

The descriptive analysis of the 13,326 care cases collected revealed the occurrence of 246 deaths, with a predominance of clinical care cases 11,514 (86.40%) in relation to traumatic care cases 1,812 (13.59%). Diseases of the circulatory system (2,384 cases; 17.88%) stood out as the main reason for care. In 2019, 6,978 (52.36%) users were served. In 2020, 6,348 (47.63%) were served. Tuesdays recorded the highest number of clinical consultations, which corresponded to 1,828 (15.87%) consultations, while Sundays had 306 (16.88%) trauma consultations. Most of the services took place in the morning, totaling 4,584 (34.39%). 

It was observed that 56.0% were emergency services, and 44.0% were transfers. Regarding the type of resource sent for assistance, the basic support unit was dispatched in 79.0% of cases and the advanced support unit in 21.0% of these.

The average age of users serviced was 51.50 years (standard deviation ±23.3), with a median of 53 years, mode of 65 years, and age range varying from 0-109 years. Women were assisted 49.60% of the time, and men 49.70%, while 0.70% did not have a record filled out regarding this information. It was observed that women comprised 52.10% of clinical care offered, while men received 64.50% of care due to traumatic causes. 

**Figure 2:**
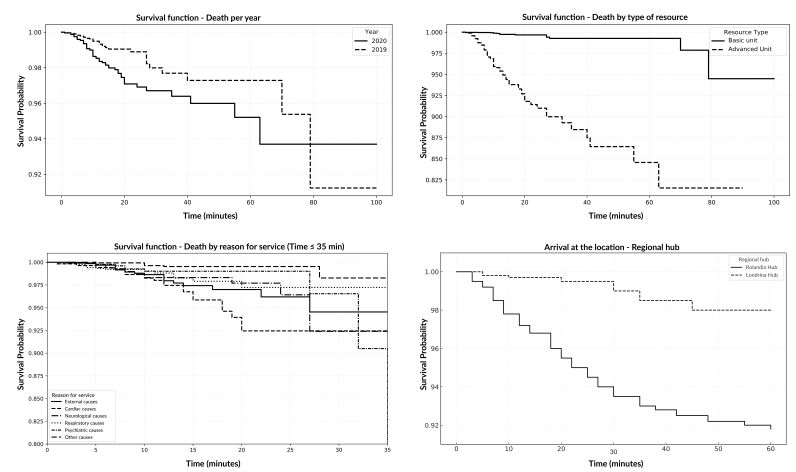
Survival curves (time-dependent), considering the time from the call to 192 until the arrival at the location of users attended by the Mobile Emergency Care Service and the outcome of death and non-death, according to sociodemographic and clinical variables. Paraná, 2024 (n=9,876)

For survival analysis, of the 13,326 calls collected, 145 were discarded because they were requests exclusively to confirm death. In 3,305 service reports, the response time variable was not fully filled out, which made it impossible to calculate the time, in minutes, between the request for service on telephone number 192 and arrival at the scene of the incident. In total, 3,450 losses were recorded, resulting in 9,876 (74.11%) cases included in the survival analysis. Of these, 101 had the outcome of death during pre-hospital care by SAMU (Table 1). Survival was analyzed using log-rank tests and Breslow’s global comparison (generalized Wilcoxon), to identify possible differences between the variables and outcomes (death, non-death) (Figure 2).

**Table 1:** Statistical summary of the Kaplan-Meier analysis (survival), considering the time from the call to 192 until arrival at the scene of the users attended by the Mobile Emergency Care Service and the outcome death and non-death, according to sociodemographic and clinical variables. Paraná, 2024 (n=9,876)

			Mean	Mantel-Cox log-rank		
Variable	Outcome	Estimate	(IC95%)	X^2^ ^a^	gl^b^	p-value
**Days of the week**	Non-death	13.48	(12.79; 14.17)	16.03	6	0.014
	Death	98.33	(97.22; 99.43)	8.84	6	0.183
**Months of the year**	Non-death	13.04	(12.78; 13.29)	11,12	11	0.432
	Death	-	-	24.33	11	0.011
Year	Non-death	13.04	(12.78; 13.29)	8.48	1	0.004
	Death	96.05	(94.58; 97.53)	12.72	1	<0.001
**Mobile resource**	Non-death	13.04	(12.78; 13.29)	0.84	1	0.359
	Death	96.05	(94.58; 97.53)	262.29	1	<0.001
**Service shift**	Non-death	13.04	(12.78; 13.29)	2.03	3	0.565
	Death	96.05	(94.57; 97.52)	2.76	3	0.430
**Age range**	Non-death	13.02	(12.67; 13.28)	10.65	14	0.713
	Death	-	-	17.26	14	0.243
Gender	Non-death	13.04	(12.78; 13.29)	0.59	1	0.442
	Death	96.16	(94.69; 97.56)	0.28	1	0.593
**Healthcare hub**	Non-death	13.04	(12.78; 13.29)	3.50	4	0.478
	Death	96.09	(94.62; -)	131.79	4	<0.001
**Reasons for service**	Non-death	13.04	(12.78; 13.29)	11.26	21	0.957
	Death	-	-	38.89	21	0.010
Nature	Non-death	13.03	(12.78; 13.29)	0.78	1	0.374
	Death	96.04	(94.57; 97.52)	2.26	1	0.133

^a^X^2^=value of the statistical test for association of variables; ^b^gl=degrees of freedom.

Regarding the reasons for care, external causes showed a significant difference in relation to the others. This showed an association with a higher risk of death during SAMU care, especially after 60 minutes, as indicated by the log-rank test (X^2^=37.40; p-value<0.001) and by Breslow comparison (X^2^=37.40; p-value<0.001), with a median response time of 9.77 minutes and interquartile analysis (IQR) of 9 among visits that resulted in death.

The analysis revealed that, in 2020, there was a higher proportion of death outcome, especially after the first 40 minutes of care. In that year, the longest median response time was observed, reaching 9.85 minutes (AIQ 9) compared to 2019, in which the time was 9.45 minutes (AIQ 9). The difference was significant for the occurrence of deaths, as demonstrated by the log-rank test (X^2^=8.21; p-value 0.004) and by the Breslow comparison (X^2^=8.11; p-value 0.004).

When considering the type of mobile resource, the basic support unit presented a median response time of 9.58 minutes (AIQ 9). The advanced support unit, responsible for assisting serious victims, had a longer time of 9.88 minutes (AIQ 9), in addition to a higher proportion of death outcome. The comparison between the type of resource and the occurrence of death was significant, as demonstrated by the log-rank test (X^2^=263; p-value<0.001) and by Breslow comparison (X^2^=263; p-value<0.001).

It is worth highlighting the occurrence of 16 cases of care resulting in death, serviced the basic support unit, all at Hub B. In these cases, the descriptive report stated that the advanced support unit was unavailable, and the victim was transported to the health service closest to the incident, for support of care and confirmation of death, an exclusive prerogative of the medical professional.

The variable related to the municipality of origin of the service, characterized by the regionalization of the service in hubs, demonstrated significant differences between Hubs A and B in the log-rank test (X^2^=132; p-value<0.001) and in Breslow’s global comparisons (X^2^=132; p-value<0.001). It was suggested that care at Hub B had a higher proportion of death outcome compared to Hub A, especially as care time increased. This characteristic could be justified based on the geographical or structural factors that affected the agility of the service. The highest number of deaths occurred in the most distant municipalities, represented by Hub B (Table 2).

**Table 2:** Summary of Pearson’s chi-square association test and death ratio (per 1,000 visits) of users dealt with by the Mobile Emergency Care Service under study, according to municipality/care center. Paraná, 2024 (n=9,876)

Hub/municipality	Outcome				
	Non-death	Death	Deaths (per 1 thousand)	X^2a^	p-value
**Hub A**					
Ibiporã	3.311	3	0.9		
Londrina	1,285	15	11.6		
**Hub B**				134.86	<0.001
Cambé	381	19	47.7		
Rolandia	4,449	47	10.5		
Others^b^	349	17	46.7		

There was a lower risk of death (HR 0.17; 95%CI 0.008; 0.37) in care not related to external causes, cardiac, neurological, respiratory, and psychiatric diseases (Table 3). It was evident that the risk of death in 2020 (HR 2.09; 95%CI 1.39; 3.16) was higher than that observed in 2019 in care provided by the advanced support unit (HR 21.51; 95%CI 12.61; 36.70), as well as in Hub B (HR 4.26; 95%CI 2.53; 7.17).

**Table 3:** Analysis of the risk of hazard ratio (HR) for death and 95% confidence interval (95%CI) by the univariate regressive model (Cox regression) between explanatory variables and occurrence of deaths in the Mobile Emergency Care Service under study. Paraná, 2024 (n=9,876)

Variable	Event		HR (95%CI) for the occurrence of death	p-value
	Non-death	Deaths		
	n	n		
**Reasons for service**				
External causes	1,485	24	-	
Cardiac	1.202	25	1.33 (0.76; 2.32)	0.324
Neurological	1.317	18	0.86 (0.47; 1.59)	0.633
Respiratory	1,352	15	0.70 (0.37; 1.34)	0.282
Psychiatric	1.053	10	0.58 (0.28; 1.22)	0.154
Others^a^	3.366	9	0.17 (0.08; 0.37)	0.001
Year				
2019	5.160	34	-	-
2020	4.615	67	2.09 (1.39; 3.16)	<0.001
**Mobile resource type**				
Basic support	7,877	16	-	-
Advanced support	1,898	85	21.51 (12.61; 36.70)	<0.001
Hubs				
A	4,585	17	-	-
B	5.175	83	4.26 (2.53; 7.17)	<0.001

^a^The other causes of death were due to neoplasia (n=3), infectious diseases (n=4) and obstetric diseases (n=2).

## Discussion

This study reinforced the importance of agility in response time of Samu to reduce mortality and, consequently, the survival of users. The prompt action of the service ensures timely care for victims of distinct health conditions, enabling necessary interventions in the face of urgent and emergency events, with a view to organizing the flow of assistance (8).

Longer response times negatively impact survival of users and compromise the results of the emergency system as a whole (10,12). This relationship was evidenced in this research, the results of which showed statistical significance when compared to the event according to the reason for service, type of resource sent and municipality/service center.

The analysis of time according to the reason for care demonstrated higher mortality in care related to diseases of the circulatory system, external and respiratory causes, all of which are time-sensitive and dependent on pre-hospital care services (13,14).

Respiratory causes represented the fourth leading cause of death in the results of this study, revealing the impact caused by the COVID-19 pandemic in the profile of reasons for care provided by SAMU, reflected by the increase in flu syndromes and respiratory failure and the decrease in trauma care (15). There was a 46.36% reduction in hospitalizations due to injuries, poisonings and other external causes between March and June 2020, compared to the same period in 2019 (16).

The findings of this research, in relation to 2020, in which the highest number of deaths and the longest response time were highlighted, corroborate international studies that found longer response times in pre-hospital services during the COVID-19 pandemic. Such evidence reinforced the importance of response time as an essential indicator for the survival of users served by SAMU and for dealing with health emergencies (17,18).

The agility of pre-hospital care is an essential component of quality care for individuals with acute illnesses and trauma throughout their life course. This enables access to timely emergency care, with a direct impact on reducing deaths and long-term disabilities (2).

This study elucidated that access to emergency care was limited in regions with lower population density and lower mobile resource coverage, especially in municipalities that make up Hub B, which presented longer response times and higher mortality rates for pre-hospital injuries. This result is consistent with research conducted in the United States, France and Spain, in which more populated areas with a greater concentration of pre-hospital resources benefited from shorter time intervals and lower mortality rates (14,19,20).

Population-based indicators are essential for analyzing the performance of emergency systems, such as SAMU, as they reflect the regionalization process, with sensitivity to diversity in epidemiological and demographic aspects, as well as in the supply and access to the service, in order to reduce mortality (10,12,13,21).

The logic of concentrating more complex resources in urban centers generates high circulation of users between health units in different locations, which increases the need for transport and results in hidden inefficiencies, such as the time spent using the ambulance for the same incident, and reduced productivity. This act was observed in this research, when analyzing the relationship between total services provided by the advanced support unit and the basic support unit. It was also observed that the response time range of advanced support, although it is a feature intended for users in greater clinical severity, presented a longer response time (3).

The high productivity of basic support reflects the Brazilian scenario, justified by the greater demand for service to low severity users and due to the need to compensate for the unavailability of advanced support. This scenario creates an overload for basic support in terms of the number of services provided and dealing with serious cases without the necessary professional prerogatives, putting healthcare safety at risk (10).

Analyzing survival and understanding the performance of SAMU regionalization was essential to identify the predictors of death, a strategic information that can help managers in planning and managing public resources allocated to structuring care systems, as well as establishing goals to reduce mortality rates and repair the infrastructure and organization conditions of this service.

This study was among the first of this magnitude in Brazil. In addition to allowing future comparisons, it offered statistics that are still scarce at a national level, which can contribute to the formulation of regionalization guidelines in other Brazilian regions and states.

As a limitation of this investigation, the incompleteness of information recorded in the pre-hospital care records stood out, a flaw frequently identified in studies on the same topic. This inconsistency arises from the urgency of other activities required in emergencies, which may compromise the accurate evaluation of the service, by causing participants to be lost to follow-up (22). This limitation did not negatively impact on this study, since the previously established sample was reached, ensuring the reliability of the results found.

The results obtained in this research made it possible to conclude that the mortality of users attended by Samu under analysis was greater in longer response time intervals, especially in time-sensitive services, such as external causes and those occurring in less populated regions, represented by Hub B. There was a longer response time and number of deaths in services conducted by advanced support in 2020. This premise confirmed the hypothesis that the process of regionalization and organization of the pre-hospital network may interfere in response time and affect the survival of the population served.

## Data Availability

The database and analysis codes used in this study are available at: https://doi.org/10.48331/scielodata.4RTE3C.
