# Supplementary material for: Analysis of mortality of users treated at a Mobile Emergency Care Service: an observational study, Paraná, 2019-2020
Source: Epidemiol Serv Saude. 2025 Sep 15;34:e20240092. doi: 10.1590/S2237-96222025v34e20240092.en (PMC12435964; doi:10.1590/S2237-96222025v34e20240092.en)
Supplement: Translation

# Translation: Análise da mortalidade de usuários atendidos em um Serviço de Atendimento Móvel de Urgência: estudo observacional, Paraná, 2019-2020

**DOI:** 10.1590/S2237-96222025v34e20240092.pt

**Authors:** Erika Fernanda dos Santos Bezerra Ludwig, Maria do Carmo Fernandez Lourenço Haddad, Mariana Ângela Rossaneis Moreira, Aroldo Gavioli, Lilia de Souza Nogueira, Eleine Aparecida Penha Martins

**Affiliations:** 1 Universidade Estadual de Londrina, Programa de Pós-Graduação em Enfermagem, Londrina, PR, Brasil; 2 Hospital Universitário Regional de Maringá, Enfermagem, Maringá, PR, Brasil; 3 Escola de Enfermagem da Universidade de São Paulo, Programa de Pós-Graduação em Enfermagem, São Paulo, SP, Brasil

**Keywords:** Mortalidade, Emergências, Serviços Médicos de Emergência, Ambulâncias, Análise de Sobrevida

## Abstract

**Objetivo:**

Analisar a mortalidade de usuários atendidos por um Serviço de Atendimento Móvel de Urgência situado no Paraná.

**Métodos:**

Trata-se de estudo transversal analítico desenvolvido a partir de relatórios de atendimento de 2019 e 2020. O Serviço de Atendimento Móvel de Urgência abrangia 21 municípios, regionalizados em polos A e B. As variáveis dependentes definidas foram óbito e tempo de atendimento. As funções de sobrevida foram calculadas pelo estimador de Kaplan-Meier e pelo teste de log-rank; a *hazard ratio* (HR) para óbito, por regressão de Cox com intervalo de confiança de 95% (IC95%).

**Resultados:**

Foram analisados 13.326 atendimentos. Destes, 246 resultaram em óbito. O risco de óbito foi maior nas solicitações sensíveis ao tempo (HR 0,17; IC95% 0,008; 0,37), em 2020 (HR 2,09; IC95% 1,39; 3,16), atendidos pelo suporte avançado (HR 21,51; IC95% 12,61; 36,70) e no polo B (HR 4,26; IC95% 2,53; 7,17).

**Conclusão:**

A mortalidade foi mais elevada em atendimentos que apresentaram intervalo de tempo prolongado para motivos sensíveis ao tempo, ocorridos nas regiões menos povoadas e atendidos pelo suporte avançado em 2020.

**Aspectos éticos**

A presente pesquisa respeitou os princípios éticos, obtendo os seguintes dados de aprovação:

Comitê de ética em pesquisa: Universidade Estadual de Londrina

Número do parecer: 4.350.880

Data de aprovação: 20/10/2020

Certificado de apresentação de apreciação ética: 39011920.1.0000.5231

Registro do consentimento livre e esclarecido: Dispensado.

## Introdução

No cenário mundial, a organização da atenção às urgências e emergências tem sido um dos desafios enfrentados por gestores de saúde (1). Em 2019, a Assembleia Mundial da Saúde destacou a relevância dos sistemas de atendimento às urgências e emergências como componente essencial da cobertura em saúde. Os países-membros foram incentivados a avaliar suas políticas e necessidades nessa área de atuação, dada sua influência determinante nos resultados de todo o sistema de saúde (2,3).

Os países que organizaram seus sistemas de atendimento às urgências tiveram impactos positivos em termos de diminuição da morbimortalidade, tempo e custo de assistência, principalmente em situações de traumas e doenças crônico-degenerativas (4,5). Esses agravos integram as linhas de cuidado prioritárias da Rede de Atenção às Urgências e Emergências do Sistema Único de Saúde e são sensíveis ao tempo, sendo influenciados pela qualidade do atendimento pré-hospitalar, ou seja, pelo Serviço de Atendimento Móvel de Urgência (Samu) (6).

Implantado em 2003 no Brasil, o Samu tem por objetivo reduzir a mortalidade, o tempo de internação e as sequelas e outras situações adversas decorrentes de intervenções tardias (7). A agilidade na resposta entre a solicitação de socorro e o atendimento aos agravos clínicos e traumáticos é considerado importante aliado à sobrevida da população atendida (8).

Apesar dos avanços do Samu, que contava com cobertura de 85,0% da população brasileira em 2022, ainda existem diversos fatores que interferem no tempo para atendimento e, consequentemente, na sobrevida das pessoas atendidas pelo serviço. Entre esses fatores, destacam-se as disparidades regionais, influenciadas pela organização da rede em saúde e pelos recursos locais (9).

Os atendimentos realizados pelo Samu no Brasil apresentaram elevação sustentada e expressiva entre 2015e 2019, superando os índices de crescimento da população coberta e dos recursos distribuídos. Este fato, também observado em outros países, está relacionado a tempos de resposta mais prolongados e ao retardo no atendimento de usuários graves, impactando negativamente a sobrevivência da população atendida e comprometendo os indicadores do sistema de saúde como um todo (10).

Diante da necessidade de promover a equidade do acesso e a qualidade da atenção ofertada pelo atendimento pré-hospitalar, este estudo tem como objetivo analisar a mortalidade de usuários atendidos por um Samu situado no interior do Paraná. Parte-se da hipótese de que o processo de regionalização e organização da rede pré-hospitalar pode interferir no tempo de resposta e afetar a sobrevivência da população atendida.

## Métodos

### Delineamento

Trata-se de estudo transversal analítico e documental, realizado entre 1º de janeiro de 2019 e 31 de dezembro de 2020, com base nos relatórios de atendimento de socorristas que prestaram atendimento em um Samu no interior do Paraná.

### Contexto

A Central de Regulação de Urgência do Samu foi implantada em 2004 e regionalizada em 2012. A central abrangia 970 mil habitantes distribuídos entre 21 municípios, organizados em dois polos de atendimento, denominados A e B. 

O polo A compreendia sete municípios, com 696.030 pessoas, dispondo de três unidades de suporte avançado, uma unidade aeromédico de asa rotativa e seis unidades de suporte básico. O polo B era representado por 14 municípios, com 273.970 pessoas, e contava com uma unidade de suporte avançado e cinco unidades de suporte básico, distribuídas em quatro bases descentralizadas (11) (Figura 1).

**Figura 1:** Distribuição de recursos móveis, unidade de suporte avançado (USA) e unidade de suporte básico (USB) do Serviço de Atendimento Móvel de Urgência em estudo, conforme regionalização em polos de atendimento. Paraná, 2024

Observaram-se a distribuição das viaturas em toda a área de extensão dos 21 municípios, a regionalização em polos A e B e a localização das bases descentralizadas com o recurso móvel disponível (Figura 1). O atendimento para suporte avançado no polo A pôde ser originário de sete municípios, os quais foram descritos a seguir, juntamente com a distância aproximada da base de atendimento, discriminada em quilômetros (km): Londrina (município com base de unidade de suporte avançado), Ibiporã (21,00 km), Jataizinho (26,70 km), Sertanópolis (45,30 km), Assaí (47,40 km), Tamarana (54,00 km) e Primeiro de Maio (69,00 km). Nesse polo, com exceção de Jataizinho, atendido pela unidade de suporte básico de Ibiporã, todos os demais contavam com base de suporte básico (11).

No polo B, as chamadas de emergência puderam originar-se de 14 municípios, entre eles: Rolândia (município com base de unidade de suporte avançado), Cambé (10,80 km), Jaguapitã (34,80 km), Prado Ferreira (37,00 km), Pitangueiras (42,80 km), Miraselva (48,70 km), Bela Vista do Paraíso (51,80 km), Guaraci (56,00 km), Florestópolis (58,60 km), Centenário do Sul (69,00 km), Porecatu (62,00 km), Alvorada do Sul (78,90 km), Lupionópolis (81,00 km) e Cafeara (87,50 km). Nesse polo, quatro dos 14 municípios possuíam base com unidade de suporte básico, sendo Rolândia responsável por atender Jaguapitã e Pitangueiras para os atendimentos com o recurso básico. Centenário do Sul prestava assistência aos demais municípios sem base própria (11).

Esse serviço operava de modo ininterrupto, 24 horas por dia. Para garantia do tempo de resposta, entendido como o intervalo entre a solicitação no número de telefone 192 e a chegada da equipe no local para o atendimento, foram imprescindíveis a articulação e a agilidade de vários profissionais. O telefonista auxiliar de regulação médica recebia as solicitações pelo número de telefone 192, anotava dados pessoais e localização da vítima e transferia a ligação ao médico regulador. Este direcionava perguntas ao solicitante que possibilitava estimar o grau de prioridade (emergência, urgência, não urgência) do atendimento, definindo o recurso móvel a ser despachado ao local da ocorrência. Subsequentemente, o rádio operador realizava o despacho por meio de contato com a equipe do recurso móvel para iniciar o deslocamento. A ação do condutor veicular era igualmente crucial, visto que devia identificar o trajeto mais rápido e seguro com vistas a salvaguardar a chegada da equipe ao local de atendimento. 

Todas essas etapas estavam expostas a possíveis falhas que poderiam comprometer o tempo de resposta, como endereço incorreto, escolha inadequada do recurso móvel de acordo com as necessidades da vítima, número insuficiente de viaturas para atender à demanda de solicitações ou definição imprópria do trajeto até o local da ocorrência (3).

### Participantes

Consideraram-se, exclusivamente, os atendimentos que geraram regulação médica e despacho de equipes para o socorro, uma vez que o preenchimento do relatório ocorreu somente nos casos em que houve deslocamento do recurso móvel. Foram excluídos os relatórios com dados incompletos e os atendimentos gerados unicamente para constatação de óbito, visto que o objetivo deste estudo consistiu em analisar a mortalidade diante da atuação do atendimento pré-hospitalar do Samu.

### Variáveis

Os dados foram coletados entre janeiro e dezembro de 2021, em conformidade com os critérios de inclusão estabelecidos. Utilizou-se instrumento elaborado pela pesquisadora por meio do Microsoft Excel. O acesso dos dados foi determinado à pesquisadora e a duas pessoas adicionais, participantes da coleta e devidamente treinadas, visando garantir a dupla verificação dos dados coletados. 

Foram coletadas as variáveis dependentes óbito e tempo de resposta. A primeira variável foi considerada durante o atendimento pré-hospitalar e categorizada em duas classes: óbito e não óbito. O tempo de resposta foi determinado a partir da solicitação no número de telefone 192 até a chegada no local para o atendimento, mensurado em minutos.

As variáveis independentes analisadas foram: sexo, idade categorizada em anos (0-11, 12-19, 20-59 e ≥60 anos), conforme preconizado pela Organização Mundial da Saúde, município de origem do atendimento, natureza do atendimento (socorro, transferência), motivo da solicitação (causas externas, cardíacas, neurológicas, respiratórias, psiquiátricas ou outros), dia da semana e turno de atendimento (manhã, tarde, noite, madrugada), tipo de equipe despachada (unidade de suporte básico, unidade de suporte avançado) e ano em que foi realizado o atendimento (2019, 2020).

### Fontes de dados e mensuração

Selecionaram-se os relatórios de atendimentos dos dois municípios que contavam com unidades de suporte avançado – recurso de maior especialização direcionado aos usuários em estado crítico, ou seja, aqueles que necessitavam de agilidade à chegada no local. Essa seleção contemplou a área de abrangência dos polos A e B, o que garantiu a análise dos 21 municípios atendidos pelo Samu. 

### Tamanho do estudo e controle de viés

O tamanho da amostra levou em consideração o número total de atendimentos realizados pelo Samu no período estudado, sendo 230.765 atendimentos com despacho de recurso ao local solicitado. Para atingir a representatividade numérica que possibilitasse a generalização, efetuou-se cálculo da amostra, por meio do programa PEPI (Programs for Epidemiologists, versão 4.0), além de fórmula para estimação de proporções, com margem de erro aceitável de 1% e intervalo de confiança de 95%, que estabeleceu amostra mínima de 9.221 relatórios de atendimento do socorrista.

Os dados foram coletados até que a amostra mínima fosse atingida. Muitos relatórios apresentavam incompletude parcial dos dados. Foram analisados 13.326 relatórios, dos quais 9.876 estavam com preenchimento completo, representando 5.199 (52,64%) atendimentos do polo A e 4.677 (47,35%) do polo B.

### Métodos estatísticos

Para a análise de dados, foi considerado o nível de significância de 5% (p-valor>0,05). As funções de sobrevida foram calculadas por meio do estimador de Kaplan-Meier, sem e com estratificação, utilizando o teste de log-rank e a comparação global de Breslow (Wilcoxon generalizado). Para a análise do risco de óbito *hazard ratio* (HR), em função do tempo, foi utilizado o modelo regressivo univariado (regressão de Cox). O teste qui-quadrado de Pearson foi utilizado para calcular a razão de óbitos (por 1 mil atendimentos) em associação com o tempo (considerado a partir da solicitação no número de telefone 192 até a chegada no local para o atendimento) e o polo de atendimento.

## Resultados

A análise descritiva dos 13.326 atendimentos coletados revelou a ocorrência de 246 óbitos, com predominância de atendimentos clínicos 11.514 (86,40%) em relação aos traumáticos 1.812 (13,59%). As doenças do aparelho circulatório (2.384 casos;17,88%) destacaram-se como a principal causa de atendimento. Em 2019, 6.978 (52,36%) usuários foram atendidos. Em 2020, foram 6.348 (47,63%). A terça-feira registrou o maior número de atendimentos clínicos, o que correspondeu a 1.828 (15,87%) atendimentos, enquanto o domingo notabilizou 306 (16,88%) atendimentos traumáticos. A maioria dos atendimentos ocorreu no período da manhã, totalizando 4.584 (34,39%). 

Observou-se que 56,0% foram atendimentos de socorro, e 44,0%, de transferências. Sobre o tipo de recurso enviado para o atendimento, 79,0% das vezes foi despachada a unidade de suporte básico e, em 21,0%, a unidade de suporte avançado.

A média de idade dos usuários atendidos foi 51,50 anos (desvio-padrão ±23,^3^), com mediana de 53 anos, moda de 65 anos, e faixa etária variando de 0-109 anos. As mulheres foram atendidas em 49,60% das vezes, e os homens, 49,70%, enquanto 0,70% não possuíam registro preenchido acerca dessa informação. Observou-se que 52,10% dos atendimentos clínicos ocorreram em mulheres, e 64,50% dos atendimentos por causas traumáticas foram identificados em homens. 

Para a análise de sobrevivência, dos 13.326 atendimentos coletados, 145 foram descartados por se tratar de solicitações para, exclusivamente, constatar óbito. Em 3.305 relatórios de atendimento, não havia o preenchimento completo da variável tempo de resposta, o que impossibilitava o cálculo do tempo, em minutos, entre a solicitação de atendimento no número de telefone 192 e a chegada no local da ocorrência. No total, contabilizou-se 3.450 perdas, resultando 9.876 (74,11%) atendimentos incluídos na análise de sobrevida. Destes, 101 apresentaram o desfecho óbito durante o atendimento pré-hospitalar do Samu (Tabela 1). Analisou-se a sobrevivência, por meio dos testes de log-rank e da comparação global de Breslow (Wilcoxon generalizado), a fim de identificar possíveis diferenças entre as variáveis e os desfechos (óbito, não óbito) (Figura 2).

**Tabela 1:** Sumário estatístico da análise de Kaplan-Meier (sobrevivência), considerando o tempo da entrada da ligação no telefone 192 até a chegada ao local de usuários atendidos pelo Serviço de Atendimento Móvel de Urgência e o desfecho óbito e não óbito, segundo variáveis sociodemográficas e clínicas. Paraná, 2024 (n=9.876)

			Média	Log-rank de Mantel-Cox		
Variável	Desfecho	Estimativa	(IC95%)	X^2^ ^a^	gl^b^	p-valor
**Dias da semana**	Não óbito	13,48	(12,79; 14,17)	16,03	6	0,014
	Óbito	98,33	(97,22; 99,43)	8,84	6	0,183
**Meses do ano**	Não óbito	13,04	(12,78; 13,29)	11,12	11	0,432
	Óbito	-	-	24,33	11	0,011
Ano	Não óbito	13,04	(12,78; 13,29)	8,48	1	0,004
	Óbito	96,05	(94,58; 97,53)	12,72	1	<0,001
**Recurso móvel**	Não óbito	13,04	(12,78; 13,29)	0,84	1	0,359
	Óbito	96,05	(94,58; 97,53)	262,29	1	<0,001
**Turno de atendimento**	Não óbito	13,04	(12,78; 13,29)	2,03	3	0,565
	Óbito	96,05	(94,57; 97,52)	2,76	3	0,430
**Faixa etária**	Não óbito	13,02	(12,67; 13,28)	10,65	14	0,713
	Óbito	-	-	17,26	14	0,243
Sexo	Não óbito	13,04	(12,78; 13,29)	0,59	1	0,442
	Óbito	96,16	(94,69; 97,56)	0,28	1	0,593
**Polo de atendimento**	Não óbito	13,04	(12,78; 13,29)	3,50	4	0,478
	Óbito	96,09	(94,62; -)	131,79	4	<0,001
**Motivos de atendimento**	Não óbito	13,04	(12,78; 13,29)	11,26	21	0,957
	Óbito	-	-	38,89	21	0,010
Natureza	Não óbito	13,03	(12,78; 13,29)	0,78	1	0,374
	Óbito	96,04	(94,57; 97,52)	2,26	1	0,133

^a^X^2^=valor do teste estatístico para associação das variáveis; ^b^gl=graus de liberdade.

**Figura 2:** Curvas de sobrevivência (tempo dependente), considerando o tempo da entrada da ligação no telefone 192 até a chegada ao local de usuários atendidos pelo Serviço de Atendimento Móvel de Urgência e o desfecho óbito e não óbito, segundo variáveis sociodemográficas e clínicas. Paraná, 2024 (n=9.876)

Em relação aos motivos de atendimento, as causas externas apresentaram diferença significativa em relação às demais. Isso evidenciou associação ao maior risco de óbito no atendimento pelo Samu, especialmente após 60 minutos, conforme indicado pelo teste de log-rank (X^2^=37,40; p-valor<0,001) e pela comparação de Breslow (X^2^=37,40; p-valor<0,001), com tempo mediano de resposta de 9,77 minutos e análise interquartílica (AIQ) de 9 entre os atendimentos que resultaram em óbito.

A análise revelou que, em 2020, houve maior proporção do desfecho óbito, especialmente após os primeiros 40 minutos de atendimento. Nesse ano, observou-se o maior tempo mediano de resposta, atingindo 9,85 minutos (AIQ 9) em comparação com 2019, em que o tempo foi 9,45 minutos (AIQ 9). A diferença foi significativa para a ocorrência de óbitos, conforme demonstrado pelo teste de log-rank (X^2^=8,21; p-valor 0,004) e pela comparação de Breslow (X^2^=8,11; p-valor 0,004).

Ao considerar o tipo de recurso móvel, a unidade de suporte básico apresentou tempo mediano de resposta de 9,58 minutos (AIQ 9). A unidade de suporte avançado, responsável pelo atendimento a vítimas graves, apresentou tempo mais elevado de 9,88 minutos (AIQ 9), além de maior proporção do desfecho óbito. A comparação entre o tipo de recurso e a ocorrência de óbito mostrou-se significativa, conforme demonstrado pelo teste de log-rank (X^2^=263; p-valor<0,001) e pela comparação de Breslow (X^2^=263; p-valor<0,001).

Vale destacar a ocorrência de 16 atendimentos com desfecho óbito, realizados pela unidade de suporte básico, todos no polo B. Nesses casos, foi constatada, em relatório descritivo, a indisponibilidade da unidade de suporte avançado, sendo a vítima transportada até o serviço de saúde mais próximo da ocorrência, para apoio no atendimento e constatação de óbito, prerrogativa exclusiva do profissional médico.

A variável relacionada ao município de origem do atendimento, caracterizada pela regionalização do serviço em polos, demonstrou diferenças significativas entre os polos A e B no teste de log-rank (X^2^=132; p-valor<0,001) e nas comparações globais de Breslow (X^2^=132; p-valor<0,001). Sugeriu-se que os atendimentos no polo B tiveram maior proporção do desfecho óbito em comparação com o polo A, especialmente à medida que o tempo de atendimento aumentava. Essa característica pôde ser justificada como reflexo dos fatores geográficos ou estruturais que afetavam a agilidade do serviço. O maior número de óbitos ocorreu nos municípios mais distantes, representados pelo polo B (Tabela 2).

**Tabela 2:** Sumário do teste de associação qui-quadrado de Pearson e razão de óbitos (por 1 mil atendimentos) de usuários atendidos pelo Serviço de Atendimento Móvel de Urgência em estudo, segundo município/polo de atendimento. Paraná, 2024 (n=9.876)

Polo/município	Desfecho				
	Não óbito	Óbito	Óbitos (por 1 mil)	X^2a^	p-valor
**Polo A**					
Ibiporã	3.311	3	0,9		
Londrina	1.285	15	11,6		
**Polo B**				134,86	<0,001
Cambé	381	19	47,7		
Rolândia	4.449	47	10,5		
Outros^b^	349	17	46,7		

^a^X^2^=valor do teste estatístico para associação das variáveis; ^b^Todos os demais municípios atendidos.

Houve menor risco de óbito (HR 0,17; IC95% 0,008; 0,37) em atendimentos não relacionados às causas externas, doenças cardíacas, neurológicas, respiratórias e psiquiátricas (Tabela 3). Evidenciou-se que o risco de óbito em 2020 (HR 2,09; IC95% 1,39; 3,^16^) foi superior ao observado em 2019 em atendimentos realizados pela unidade de suporte avançado (HR 21,51; IC95% 12,61; 36,70), assim como no polo B (HR 4,26; IC95% 2,53; 7,^17^).

**Tabela 3:** Análise do risco de óbito *hazard ratio* (HR) e intervalo de confiança de 95% (IC95%) pelo modelo regressivo univariado (regressão de Cox) entre variáveis explanatórias e ocorrência de óbitos no Serviço de Atendimento Móvel de Urgência em estudo. Paraná, 2024 (n=9.876)

Variável	Evento		HR (IC95%) para ocorrência de óbito	p-valor
	Não óbito	Óbitos		
	n	n		
**Motivos de atendimento**				
Causas externas	1.485	24	-	
Cardíacas	1.202	25	1,33 (0,76; 2,32)	0,324
Neurológicas	1.317	18	0,86 (0,47; 1,59)	0,633
Respiratórias	1.352	15	0,70 (0,37; 1,34)	0,282
Psiquiátricas	1.053	10	0,58 (0,28; 1,22)	0,154
Outros^a^	3.366	9	0,17 (0,08; 0,37)	0,001
Ano				
2019	5.160	34	-	-
2020	4.615	67	2,09 (1,39; 3,16)	<0,001
**Tipo de recurso móvel**				
Suporte básico	7.877	16	-	-
Suporte avançado	1.898	85	21,51 (12,61; 36,70)	<0,001
Polos				
A	4.585	17	-	-
B	5.175	83	4,26 (2,53; 7,17)	<0,001

^a^As outras causas de óbito ocorreram por neoplasia (n=3), doenças infectocontagiosas (n=4) e obstétricas (n=2).

## Discussão

Este estudo reforçou a importância da agilidade no tempo de resposta do Samu para a redução da mortalidade e, consequentemente, a sobrevida dos usuários. A pronta atuação do serviço garante atendimento precoce às vítimas de agravos à saúde de natureza distintas, viabilizando intervenções necessárias diante de eventos de urgência e emergência, com vistas a ordenar o fluxo assistencial (8).

Tempos de resposta mais longos impactam negativamente a sobrevivência dos usuários e comprometem os resultados do sistema de emergência como um todo (10,12). Essa relação foi evidenciada nesta pesquisa, cujos resultados apontaram significância estatística quando comparados ao evento de acordo com o motivo de atendimento, tipo de recurso enviado e município/polo de atendimento.

A análise do tempo em função do motivo de atendimento demonstrou maior mortalidade em atendimentos relacionados às doenças do aparelho circulatório, causas externas e respiratórias, todas sensíveis ao tempo e dependentes do serviço de atendimento pré-hospitalar (13,14).

As causas respiratórias representaram a quarta causa de morte nos resultados deste estudo, revelando o impacto causado pela pandemia da covid-19 no perfil dos motivos de atendimento realizados pelo Samu, refletido pelo aumento das síndromes gripais e insuficiência respiratória e pelo decréscimo dos atendimentos por trauma (15). Houve redução de 46,36% nas internações por lesões, envenenamentos e outras causas externas entre março e junho de 2020, em comparação ao mesmo período de 2019 (16).

Os achados desta pesquisa, em relação a 2020, em que foram destacados o maior número de óbitos e o maior tempo de resposta, corroboram estudos internacionais que constataram tempos de resposta mais longos nos serviços pré-hospitalares durante a pandemia da covid-19. Tais evidências reforçaram a importância do tempo de resposta como indicador essencial para a sobrevida dos usuários atendidos pelo Samu e enfrentamento às emergências sanitárias (17,18).

A agilidade do atendimento pré-hospitalar é componente essencial de qualidade da assistência a indivíduos com doenças agudas e traumas em todo o curso da vida. Isso possibilita o acesso ao cuidado emergencial em tempo oportuno, com implicação direta na redução de mortes e incapacidades a longo prazo (2).

Este estudo elucidou que o acesso ao cuidado emergencial se mostrou limitado para as regiões de menor densidade populacional e com menor cobertura de recursos móveis, especialmente nos municípios que compõem o polo B, os quais apresentaram tempo de resposta mais elevado e maiores taxas de mortalidade para agravos pré-hospitalares. Esse resultado é compatível com pesquisas realizadas nos Estados Unidos, na França e na Espanha, em que as áreas mais povoadas e com maior concentração de recursos pré-hospitalares se beneficiaram de intervalos de tempo mais curtos e menores taxas de mortalidade (14,19,20).

Indicadores de base populacional são fundamentais para análise de desempenho de sistemas de emergência, como o Samu, pois refletem o processo de regionalização, com sensibilidade à diversidade nos aspectos epidemiológicos e demográficos, bem como na oferta e no acesso do serviço, a fim de reduzir a mortalidade (10,12,13,21).

A lógica de concentrar recursos de maior complexidade nos centros urbanos gera alta circulação de usuários entre unidades de saúde de diferentes localidades, o que aumenta a necessidade de transportes e culmina em ineficiências ocultas, como o tempo de utilização da ambulância na mesma ocorrência, e redução da produtividade. Esse fato foi observado nesta pesquisa ao analisar a relação de atendimentos totais realizados pela unidade de suporte avançado e pela unidade de suporte básico. Observou-se também que o intervalo de tempo de resposta do suporte avançado, embora seja um recurso destinado a usuários em maior gravidade clínica, apresentou tempo de resposta mais elevado (3).

A alta produtividade do suporte básico reflete o cenário brasileiro, justificada pela maior demanda de atendimento aos usuários de baixa gravidade e devido à necessidade de compensar a indisponibilidade do suporte avançado. Esse cenário gera sobrecarga para o suporte básico no que diz respeito ao quantitativo de atendimentos e ao enfrentamento a casos graves sem as prerrogativas profissionais necessárias, colocando em risco a segurança assistencial (10).

Analisar a sobrevida e conhecer o desempenho da regionalização do Samu foi primordial para identificar os fatores preditores de óbito, informações estratégicas que podem auxiliar os gestores no planejamento e na gestão dos recursos públicos destinados à estruturação dos sistemas de atendimento, bem como estabelecer metas para redução das taxas de mortalidade e reparo nas condições de infraestrutura e organização deste serviço.

Este estudo figurou entre os primeiros com essa magnitude no Brasil. Além de permitir comparações futuras, ofereceu estatísticas ainda escassas em nível nacional, as quais podem contribuir para a formulação das diretrizes de regionalização em outras regiões e estados brasileiros.

Como limitação desta investigação, destacou-se a incompletude de informações registradas nas fichas de atendimento pré-hospitalar, uma falha frequentemente identificada em estudos na mesma temática. Essa inconsistência decorre da premência de outras atividades a serem realizadas nas emergências, o que pode comprometer a avaliação precisa do serviço, por ocasionar perdas de seguimento dos participantes (22). Essa limitação não impactou negativamente este estudo, uma vez que a amostra previamente estabelecida foi atingida, garantindo a confiabilidade dos resultados encontrados.

Os resultados obtidos nesta pesquisa possibilitaram concluir que a mortalidade dos usuários atendidos pelo Samu em análise foi maior em intervalos de tempos de resposta mais longos, sobretudo nos atendimentos sensíveis ao tempo, tais quais as causas externas e os ocorridos em regiões menos povoadas, representadas pelo polo B. Verificou-se maior tempo de resposta e número de óbitos nos atendimentos conduzidos pelo suporte avançado em 2020. Essa premissa confirmou a hipótese de que o processo de regionalização e organização da rede pré-hospitalar pode interferir no tempo de resposta e afetar a sobrevivência da população atendida.

## Data Availability

O banco de dados e os códigos de análise utilizados na pesquisa estão disponíveis em: https://doi.org/10.48331/scielodata.4RTE3C.
